# Development and evaluation of psychoeducational resources for adult carers to emotionally support young people impacted by wars: A community case study

**DOI:** 10.3389/fpsyg.2022.995232

**Published:** 2022-11-02

**Authors:** Giada Vicentini, Roberto Burro, Emmanuela Rocca, Cristina Lonardi, Rob Hall, Daniela Raccanello

**Affiliations:** ^1^Department of Human Sciences, University of Verona, Verona, Italy; ^2^Environmetrics Pty Ltd, Killara, NSW, Australia; ^3^Macquarie University, Sydney, NSW, Australia

**Keywords:** communication campaigns, wars, victims, emotions, coping strategies, children, adolescents, psychoeducation

## Abstract

Wars and armed conflicts have a devastating impact at the economic, social, and individual level. Millions of children and adolescents are forced to bear their disastrous consequences, also in terms of mental health. Their effects are even more complicated when intertwined with those of other disasters such as the current COVID-19 pandemic. To help them face such adverse events, lay adults can be supported by psychoeducational interventions involving simple tools to assist children and adolescents emotionally. Hence, we planned and implemented two public communication campaigns concerning wars to support adult carers such as parents, teachers, educators, psychologists, first responders, and others interested in young people’s wellbeing. We developed psychoeducational materials to help children and adolescents cope with negative emotions related to indirect and direct exposure to wars. This study had the objective to identify the content for two pamphlets, testing their comprehensibility, usability, and utility, and monitoring their dissemination. First, based on classifications of coping strategies and on a previous campaign about COVID-19 pandemic, we decided to include in the psychoeducational materials basic information on news about wars and common reactions to wars, respectively; on emotions that might be experienced; and on coping strategies for dealing with negative emotions. For the first pamphlet, we identified the strategies involving 141 adults. They completed an online survey with open-ended questions concerning ways to help children and adolescents cope with negative emotions associated with the Russia-Ukraine war. For the second pamphlet, we selected the contents based on Psychological First Aid manuals. Through content analyses, we chose 24 strategies. Second, data gathered with 108 adults who had consulted the psychoeducational materials supported their comprehensibility, usability, and utility. Third, we monitored the visibility of the campaigns after the release of the pamphlets, using Google Analytics™ data from the HEMOT^®^ website through which we disseminated them. To conclude, our findings supported the comprehensibility, the usability, and the utility of the two pamphlets, to be disseminated as psychoeducational materials in the early phase of a disaster.

## Introduction

Wars and armed conflicts, such as the Russia-Ukraine war, have a devastating impact at the economic, social, and individual level. Millions of children and adolescents are killed, injured, mutilated, orphaned, or uprooted from their homes. Several studies have documented the traumatic effects for children and adolescents’ mental health (Dimitry, 2012; Betancourt et al., 2013; Slone and Mann, 2016; Vossoughi et al., 2018; Aghajafari et al., 2020; Jin et al., 2021). Yet, young people can also demonstrate resilience, showing positive psychological adaptation (Masten, 2021). Lay adults can be coached to use simple psychoeducational interventions to assist children and adolescents build resilience (Galea et al., 2020; Horesh and Brown, 2020; Raccanello et al., 2020; Xiang et al., 2020).

Hence, we implemented two public communication campaigns to give adults involved with children and adolescents, tools to assist young people cope with negative emotions triggered by indirect and direct exposure to war. While direct victims suffer immediate physical and emotional damage, indirect victims are affected over time and at a distance from the physical trauma. The indirect impact can be mediated by social or mass media exposure (UNDRR, n.d.).

The campaigns were developed within the Centre of Research in Psychology HEMOT® (Helmet for EMOTions) at the University of Verona’s Department of Human Sciences.

## Background and rationale

According to the Diagnostic and Statistical Manual of Mental Disorders (DSM-5; APA, 2013), wars and armed conflicts can have traumatic consequences more severe than from other disasters, being caused by intentional and voluntary actions carried out by other people. Supporting this, a meta-analysis revealed higher rates of depression for children and adolescents experiencing disasters with interpersonal violence compared to other disasters (Vibhakar et al., 2019). Overall, children and adolescents exposed to war can show a variety of traumatic symptoms. A review of studies about direct exposure to Middle East wars reported the presence of post-traumatic stress disorder (PTSD), depression, anxiety, and behavioral problems (Dimitry, 2012). Some of these symptoms were more frequent for older males, probably due to their higher exposure to the external world compared to females and younger children. Similar findings emerge from a meta-analysis considering more than 4,000 child-soldiers, showing both internalizing and externalizing symptoms (Betancourt et al., 2013). However, in this case the recruitment age was negatively correlated with symptoms, suggesting a higher risk at lower ages. Another meta-analysis identified the presence of psychosomatic symptoms (e.g., stomachache, bowel irregularity), emotional disturbances (e.g., separation anxiety, new phobias), psychopathologic symptoms (e.g., PTSD, post-traumatic symptoms), sleeping disorders (e.g., insomnia, nightmares), and behavioral problems (e.g., aggressiveness, regression) with varying rates amongst children younger than six exposed to wars and terrorism (Slone and Mann, 2016).

Other reviews confirmed the high risk of mental health problems amongst young people. Vossoughi et al. (2018) identified a variety of symptoms for three to 22-year-olds residing in refugee camps. These included somatic symptoms (e.g., headache, exhaustion, stomachache), PTSD, anxiety, depression, and aggressive behaviors. Symptoms were more frequent amongst older refugees, again perhaps because of longer exposure to adverse circumstances. Similarly, five to 12-year-old refugees showed more symptoms of psychosocial, emotional, and behavioral problems than the rest of the population (Aghajafari et al., 2020); Rates of depression and suicide have also been reported higher for child and adolescent refugees (Jin et al., 2021).

Meta-analytic studies have also demonstrated the role of parents in moderating traumatic effects on children (Dimitry, 2012; Slone and Mann, 2016; Vossoughi et al., 2018; Aghajafari et al., 2020; Eltanamly et al., 2021; Jin et al., 2021). In the context of war, Eltanamly et al. (2021) showed that a parent’s educational style lacking warmth and gentleness correlated positively with children showing symptoms such as depression, anxiety, or social problems, and negatively with adjustment indicators such as self-esteem, school achievement, or prosocial behaviors. Levels of depression between mothers and offspring were shown to be correlated (Dimitry, 2012). For children younger than six years, disturbances were linked to parental psychopathology and poor family functioning (Slone and Mann, 2016). Amongst refugees living in camps, there were correlations between levels of psychopathology shown by youths and their mothers (Vossoughi et al., 2018). Parents’ psychological health was identified as a risk factor for the development of psychopathology in five to nine-year-old refugees (Aghajafari et al., 2020). Finally, being accompanied by at least one family member had a protective role against depression and suicide amongst minors (Jin et al., 2021).

When people confront disasters and wars, they are forced to activate coping processes. Such cognitive, emotional, and behavioral strategies help to diminish the impact of an event which is or is perceived as traumatic (Lazarus and Folkman, 1984). Zimmer-Gembeck and Skinner (2011) identified 12 families of strategies grouped into three categories (Table 1), linked to Deci and Ryan’s (1985) three basic needs for competence, relatedness, and autonomy. Each category includes two adaptive and two maladaptive families of coping strategies, that can be activated when people perceive an event as a challenge or threat (for family definitions and disaster-related applications see Burro et al., 2021; Raccanello et al., 2020, 2021a, 2022b). Adaptive strategies are, for competence: problem solving, information seeking; for relatedness: self-reliance, support seeking; for autonomy: accommodation, negotiation. Maladaptive strategies are, for competence: helplessness, escape; for relatedness: delegation, social isolation; for autonomy: submission, opposition.

**Table 1:** Families of coping strategies (Zimmer-Gembeck and Skinner, 2011).

Basic needs	Coping strategies	Label used in the pamphlet	Examples	*N* (%)	*M* (*SD*)	95% CI
Competence	Problem solving	Take steps to solve the problem	Asking “What can you do to change this situation?”	105 (11.3%)	0.75 (1.18)	0.55–0.94
			Beginning to build peace in your life.			
	Information seeking	Talk about facts	Searching for what we do not understand or deepen unclear news.	119 (12.9%)	0.84 (1.17)	0.65–1.04
			Giving news about the war, in different ways to a child or an adolescent.			
	Helplessness	–	Tragedy is part of life.	3 (0.3%)	–	–
			Insecurity.			
	Escape	–	Avoiding following news addressed to adults.	13 (1.4%)	–	–
			Imagining a different world.			
Relatedness	Self-reliance	Understand and express your emotions	It is normal to feel sadness, fear, anger.	227 (24.5%)	1.61 (1.89)	1.30–1.93
			Drawing or writing how you feel.			
	Social support	Receive and give help	We can find together something to do for helping children that are in war.	236 (25.5%)	1.67 (1.60)	1.41–1.94
			Spending a lot of time with people you love.			
	Delegation	–	–	–	–	–
	Social isolation	–	–	–	–	–
Autonomy	Accommodation	Take some time to focus on other things	Organizing the day filling it with activities and games.	207 (22.4%)	1.47 (1.74)	1.18–1.76
			Thinking about happy things, that make you feel well.			
	Negotiation	Adapt	Changing routines.	8 (0.9%)	–	–
			Sometimes during difficult situations we appreciate more those important things that we had taken for granted before.			
	Submission	–	–	–	–	–
	Opposition	–	Adults’ world is complicated and the greedy people want money and power.	7 (0.8%)	–	–
			Conflicts exist also between children: The problem is that often adults do not learn how to manage them.			

We reported basic needs, families of coping strategies, labels used in the pamphlet, examples from the survey for developing the first pamphlet, number, N (and percentage, %), means, M (and standard deviation, SD), and 95% confidence intervals, CI (these data are reported only for the families included in the GLMM).

A meta-analysis indicated that, for child and adolescent victims of natural disasters, problem solving, support seeking, and submission have an adaptive role, while escape, social isolation, submission, and opposition are maladaptive (Raccanello et al., 2022a). A similar pattern was shown for COVID-19 pandemics where escape and social isolation were linked to traumatic symptoms, while problem solving, self-reliance, social support, and accommodation were related to adaptation (Raccanello et al., 2021b). During armed conflicts, as cited above, both family and community social support are protective factors (Betancourt et al., 2013; Aghajafari et al., 2020). Similarly, restoring access to educational opportunities, including schools, can be another key factor for protecting children and adolescents (Betancourt et al., 2013; Aghajafari et al., 2020).

Promoting adaptive coping strategies can give children and adolescents relevant resources to cope with the different phases of a disaster. Being able to recognize and understand emotions, and the ability to regulate them form what is called emotional competence, which plays a key role for adapting to a social context (Denham, 1998). Enhancing young people’s emotional competence can be a major step in preparing them to deal with potentially damaging life events. Adults can be coached to assist children by being supplied with basic information about the nature of traumatic events, common reactions to the events, possible stress symptoms, and coping strategies (Hisli Sahin et al., 2011). As an example, Raccanello et al. (2020) conducted a public communication campaign to support adults in helping young people cope with negative emotions associated with the COVID-19 pandemic.

There are also other forms of psychological support that can be implemented when an emergency occurs. One is Psychological First Aid (PFA), an evidence-informed approach (Birkhead and Vermeulen, 2018) comprising eight core actions: contact and engagement, safety and comfort, stabilization, information gathering, practical assistance, connection with social supports, information on coping, and linkage to collaborative services (NCTSN and NCPTSD, 2006). In many cases the efficacy of this and other interventions following disasters is higher when parents are involved (Kramer and Landolt, 2011; Newman et al., 2014; Gutermann et al., 2016; McGuire et al., 2021).

This study describes a community case study (Smith et al., 2016) about the development and distribution of material designed to help adults support young people dealing with negative emotions linked to experiences of war. Through the process described in this study, we documented how it is possible to make available to a large audience psychoeducational materials using an evidence-informed approach (Birkhead and Vermeulen, 2018). This is particularly relevant in case such materials refer to coping strategies, whose level of efficacy depends on a variety of individual and contextual factors. The materials consist of two pamphlets relevant to indirect exposure (first pamphlet) and direct exposure (second pamphlet) to war. We had three aims:

1. Creating the content of the pamphlets using a variety of methodologies, such as studying previous campaigns (e.g., Raccanello et al., 2020) and established classifications of coping strategies (e.g., Zimmer-Gembeck and Skinner, 2011), applying content analysis to the responses of lay adults, and coding the contents of informative material such as PFA manuals (NCTSN and NCPTSD, 2006; World Health Organization et al., 2011).
2. Testing the comprehensibility, the usability, and the utility of the pamphlets across two adult samples.
3. Monitoring the dissemination of the pamphlets, using Google Analytics™ data of the HEMOT® website, from which they could be downloaded.

## Case description

According to the United Nations High Commissioner for Refugees (UNHCR, 2022a), at the end of 2021 there were about 89.3 million people worldwide who had been displaced by armed conflicts or disasters. In June 2022, there were at least 27 active conflicts (e.g., civil wars, territorial disputes, political instabilities; Center for Prevention Action, 2022). Among these conflicts there was the Russia-Ukraine war, begun on 24^th^ February 2022. Until June 2022, there were more than 4,000,000 Ukrainian refugees recorded across Europe (UNHCR, 2022b). Italy welcomed more than 125,000 people, including many children and adolescents. It is difficult to estimate the true primarily extent of the negative effects of this conflict because it is both ongoing and intertwined with disasters such as the current COVID-19 pandemic we are facing, with cascading and cumulative traumatic effects (Slone and Mann, 2016).

### Partnerships

The campaigns were implemented by a multi-disciplinary team including experts in psychology and sociology (HEMOT® Centre) and conducted in collaboration with the University of Verona. The second campaign involved also external partners, i.e., the Civil Protection of the Veneto Region and the Order of Psychologists of Veneto, managing technical and psychological aspects of the emergency at a regional level. Logos of supporting organizations are included in the pamphlets.

## Materials and methods

### Target setting and population

For each campaign, we developed a pamphlet addressed to adults such as parents, teachers, educators, psychologists, and others interested in young people’s wellbeing. The second pamphlet was primarily addressed to first responders who must manage the reception of refugees. Both pamphlets were released during the spring 2022 in Northern Italy and then disseminated through a variety of media.

The first pamphlet was initially available in Italian and English, and then made available in 23 languages including also Albanian, Amharic/Ethiopian, Arabic, Bahasa Melayu, Bengali, Croatian, Finnish, French, Greek, Hungarian, Indonesian, Lugbara, Mandarin, Portuguese, Romanian, Russian, Slovenian, Spanish, Swahili, Swedish, and Ukrainian.

We then released the second pamphlet in Italian, English, Ukrainian, and Russian for Italian first responders dealing with foreign refugees. Subsequently, we released versions in Albanian, Arabic, Bahasa Melayu, Croatian, Mandarin, Persian, Portuguese, Romanian, and Spanish, for a total of 13 languages.

### Campaign content

**Figure 1:**
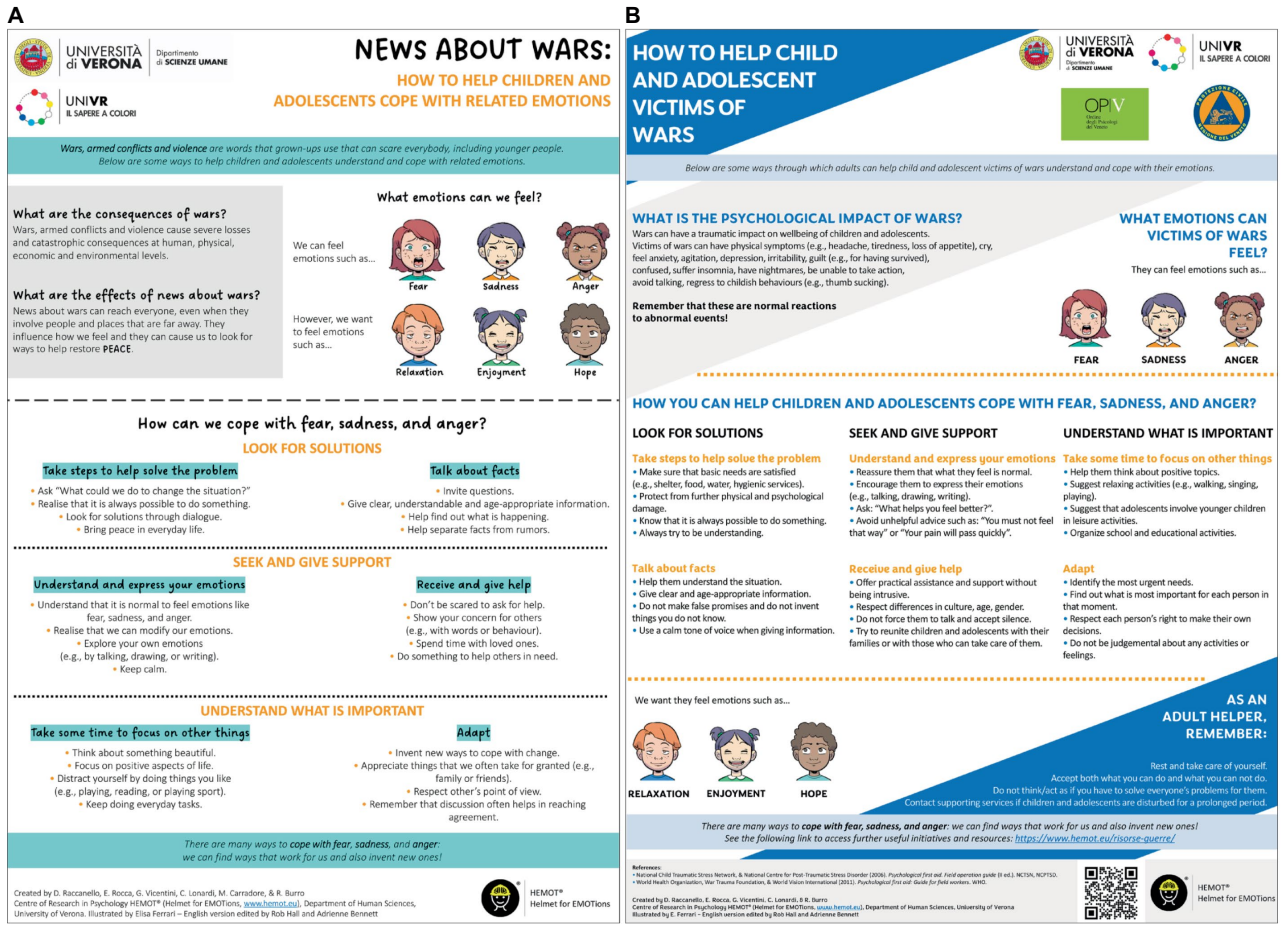
The English version of **(A)** the pamphlet for supporting adults to help children and adolescents cope with news about wars, and **(B)** the pamphlet for supporting adults to help children and adolescent victims of wars.

Each pamphlet was divided into three main sections (Figure 1), as in the previous campaign on COVID-19 pandemic (Raccanello et al., 2020). Each part was introduced by titles containing questions represented with the same formatting style, i.e., black bold for the first pamphlet and upper case blue bold for the second pamphlet. We present briefly the main contents and the graphical characteristics of the corresponding sections in the following summary.

1. The first section described some characteristics of the disaster at issue, i.e., wars and their consequences. Graphically, this information was represented within a grey box in the first pamphlet and within a grey oblique box in the second pamphlet.
2. The second section focused on emotions associated with wars. Graphically, it comprised the parts with the drawings of faces expressing emotions.
3. The third section included information about coping strategies. Graphically, this part was inserted in the central part of the pamphlets.

As follows, we describe in details the contents of the two pamphlets. In both of them, we referred to the emergency context and to the targets in the titles.

The first section provided general information about the impact of wars and reporting of wars (Pfefferbaum et al., 2019) for the first pamphlet, and a list of traumatic consequences for minors directly experiencing wars (World Health Organization et al., 2011) for the second pamphlet.

The second section (split in two subsections for the second pamphlet) reported verbal labels and drawings about negative emotions caused by exposure to wars and news about wars, i.e., *fear*, *sadness*, and *anger* (Dalgleish and Power, 2004; Raccanello et al., 2017, 2021a), and positive emotions to be promoted, i.e., *relaxation*, *enjoyment*, and *hope*. We choose to insert some basic emotions (i.e., fear, sadness, anger, enjoyment) for which there is a universal correspondence between labels and facial expressions (Ekman, 1992, 1993); we also included other emotions, i.e., relaxation and hope, for their relevance in this context.

The third section focused on coping strategies, using Zimmer-Gembeck and Skinner’s (2011) classification as the main theoretical framework and adapting the contents from the COVID-19-related pamphlet (Raccanello et al., 2020). To identify the coping strategies for the first pamphlet, we conducted an online survey.

1. *Participants*. We involved a sample of lay adults. Selection criteria included age (being older than 18 years) and understanding Italian for completing the survey. We sent the link to graduate and undergraduate university students attending the University of Verona, Italy. We asked them to share the link with other adults, using a snowball sampling method. Participants gave their informed consent for data collection and treatment (approval of the Ethical Committee of the Department of Human Sciences, University of Verona, protocol number 159372).
2. *Materials and procedure*. We administered an open-ended question (*What would you say to a child or an adolescent to help him/her cope with the negative emotions (e.g., sadness, fear, anger) due to the news about the ongoing Russia-Ukraine war?*) followed by 10 spaces. A first judge coded all the answers counting the number of responses corresponding to Zimmer-Gembeck and Skinner’s (2011) 12 families. A second judge coded the 30% for reliability (accordance rate: 98%).

To select strategies for the second pamphlet, we adapted some of those inserted in the first pamphlet and identified others from PFA manuals (NCTSN and NCPTSD, 2006; World Health Organization et al., 2011), coding them following Zimmer-Gembeck and Skinner’s (2011) taxonomy.

For the second pamphlet, we also added some final tips that adult carers could consider for maintaining their own wellbeing (World Health Organization et al., 2011).

At the end of each pamphlet, we included an invitation for people to suggest additional approaches for supporting young people. We did this to provide an opportunity for feedback, given that the efficacy of strategies can vary over time and across contexts. For the second pamphlet, there were also a link and a QR code to a webpage^1^ with resources for sustaining direct victims of wars – with a special attention to Ukraine as suggested by the research partners.

### Comprehensibility, usability, and utility of the campaign message

We evaluated the comprehensibility, the usability, and the utility of the pamphlets through two online surveys.

1. *Participants*. We recruited two samples of lay adults. We sent the links to graduate and undergraduate university students attending the University of Verona, Italy. We asked them to share them with other adults, using a snowball sampling method. Participants gave their informed consent to data collection and treatment (approval of the Ethical Committee of the Department of Human Sciences, University of Verona, protocol number 159372).
2. *Materials and procedure*. For each pamphlet, we asked four questions (Atkin and Freimuth, 2013; Raccanello et al., 2020), for assessing their comprehensibility (*How comprehensible are the contents of the pamphlet?*), usability (*How easy to consult are the contents of the pamphlet?*), and utility (*How useful is the message conveyed by the pamphlet for you? How useful is the message conveyed by the pamphlet for children and/or adolescents?*) each rated on a 5-point scale (1 = *not at all*, 5 = *very much*).

### Campaign dissemination

The two campaigns were disseminated through the HEMOT® Centre website^2^ and then *via* other channels.

The first pamphlet was published on 28^th^ March 2022.^3^ The following day, it was publicized by the University of Verona (e.g., UnivrMagazine, Facebook, Instagram) and by the School Office of the Veneto Region, reaching every school at a regional level. We also encouraged those who helped us with translation of the pamphlet in different languages to disseminate it using formal and informal networks, leading, for example, on 21^st^ May 2022, to the citation of the Spanish version within a YouTube video.

The second pamphlet was released on 19^th^ May 2022.^4^ The following day, the School Office of the Veneto Region published a post for downloading it. On 24^th^ May 2022, it was disseminated by the media of the University of Verona.

To monitor the dissemination of the campaigns we used data from Google Analytics™ associated with the HEMOT® website. Such data does not contain personally identifiable information (Kirk et al., 2012). For each webpage, we assessed: (a) The number of pageviews from the date of the first publication to the 30^th^ of June 2022; (b) The continent from which the pageviews originated; (c) How the visitors arrived at the webpage: direct traffic (i.e., directly to the webpage), referral traffic (i.e., through links at other websites), and organic traffic (i.e., after a search engine query).

### Data analyses

For the first pamphlet, we explored which families were more frequent, investigating differences according to respondent characteristics like gender and contact with minors (being parent of a child/adolescent and/or having a work in contact with them). We excluded from the analysis the families with a number of responses lower than 10% (i.e., negotiation and all the maladaptive ones). Using R (R Core Team, 2022), we conducted a Generalized Linear Mixed Model (GLMM) with participants as the random factor; gender (male, female) and contact with minors (no, yes) as fixed between-subject factors; family (problem solving, information seeking, self-reliance, social support, accommodation) as the fixed within-subject factor; and the number of responses for each family as the dependent variable. We implemented a model selection procedure to test whether the more complex models were significantly better than the simpler ones. Model 1 included the fixed factors; in model 2 we added the interactions between the within-subject factor and the between-subject factors; and model 3 included the three factors and both two-way and three-way interactions. We calculated seven fit indexes and considered as the target score a composite performance score, obtained by normalizing the indexes and considering the mean value for each model (Table 2). This score ranges from 0 to 100%; higher values indicate a better model performance (Burnham and Anderson, 2002; Lüdecke et al., 2021). We used the Bonferroni correction for post-hoc tests (*p* < 0.05).

**Table 2:** Fit indexes for the tested models.

Models	AIC weights	BIC weights	Conditional *R^2^*	Marginal *R^2^*	ICC	RMSE	Sigma	Performance score
Model 3. Gender + contact with minors + family + (gender^*^contact with minors) + (gender^*^family) + (contact with minors^*^family) + (gender^*^contact with minors^*^family)	0.773	< 0.001	0.365	0.197	0.209	1.345	1.000	66.67%
Model 2. Gender + contact with minors + family + (gender^*^family) + (contact with minors^*^family)	0.224	< 0.001	0.350	0.164	0.223	1.347	1.000	55.44%
Model 1. Gender + contact with minors + family	0.004	1.000	0.322	0.129	0.221	1.369	1.000	32.12%

The symbol + indicates adding a parameter; the symbol ^*^ indicates the interaction between parameters. Gender (males, females); contact with minors (no, yes); family (problem solving, information seeking, self-reliance, social support, accommodation). AIC, Akaike Information Criterion; BIC, Bayesian Information Criterion; *R*^2^, coefficient of determination; ICC, Intra-class Correlation Coefficient; RMSE, Root Mean Squared Error. For each model, the performance score is a composite score obtained by normalizing the previous seven indexes and calculating their mean value. The models are ordered according to their performance score.

We then reported and commented the descriptive statistics concerning responses about comprehensibility, usability, and utility of the pamphlets. Finally, for the data about the monitoring of the campaigns, we reported the descriptive statistics concerning the indicators from the Google Analytics™ related to the HEMOT® website.

## Results

### Sociodemographic characteristics of the participants

For the first survey (i.e., aiming at defining the contents for the first campaign), we coded responses from 141 lay adults (*M_age_* = 39.8, *SD* = 11.6; 76% females). Forty-four percent of the sample were parents of minors and the 50% had a professional role in contact with minors.

For the other two surveys (i.e., aiming at assessing the comprehensibility, the usability, and the utility of the pamphlets), we involved two samples of, respectively, 54 (*M_age_* = 34.1, *SD* = 11.5; 65% females) and 54 adults (*M_age_* = 38.7, *SD* = 15.4; 76% females). Parents of minors represented 22.6 and 35.2% of each sample, while 55.6 and 16.7% had professional roles in contact with minors.

### Developing the campaign contents

**Figure 2:**
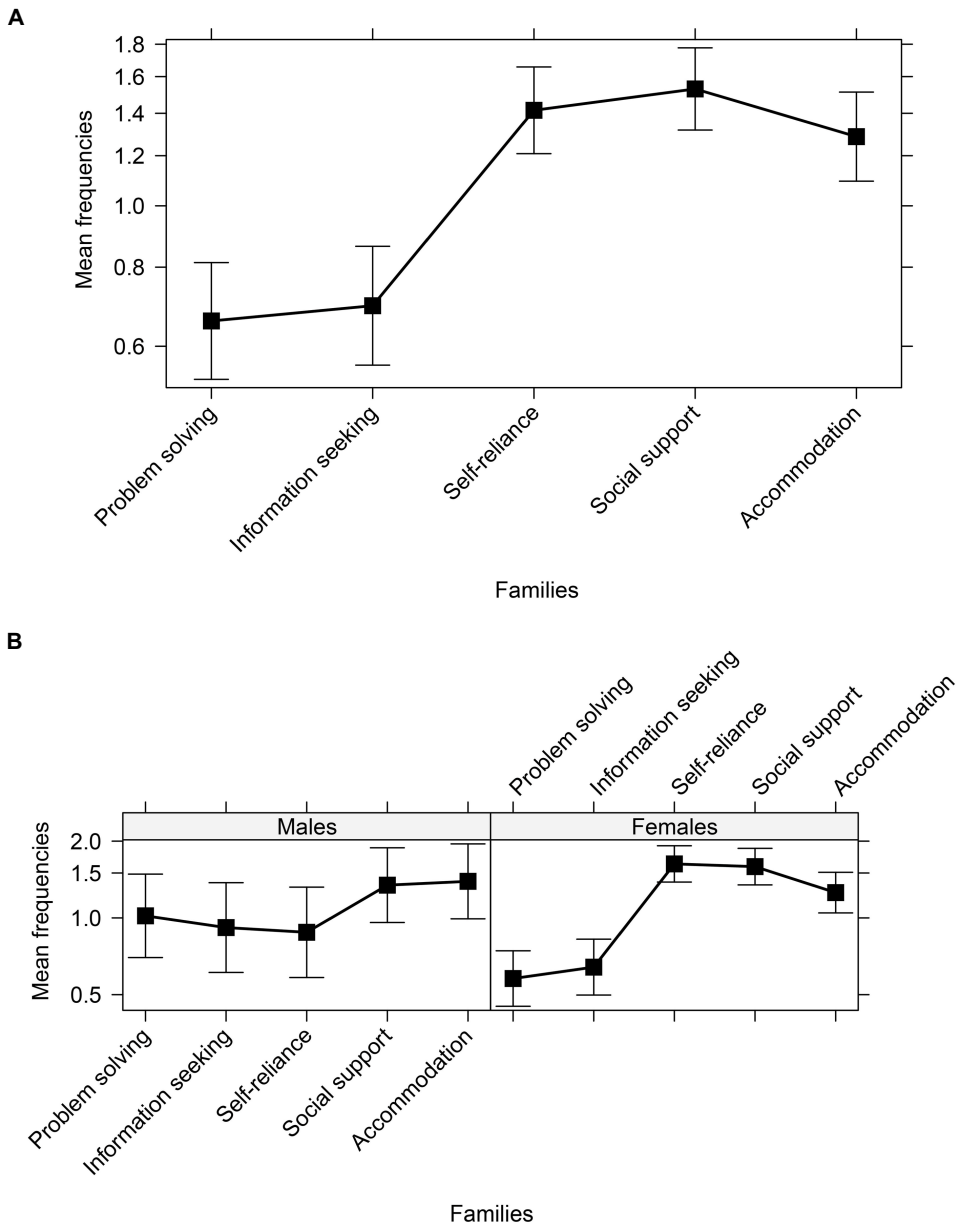
Families of coping strategies as coded in the survey of lay adults, **(A)** for the whole sample, and **(B)** separately by males and females. The bars represent the 95% CI.

For the first campaign, we obtained from the survey 925 plausible strategies (see Table 1 for examples and descriptive statistics for each family). Through the model selection procedure, we chose model 3, which revealed a significant effect of family and two significant interactions, gender × family, and gender × contact (Table 3). Post-hoc tests (Table 3) indicated that the families that were more frequently cited were social support, self-reliance, and accommodation, reported more than information seeking and problem solving (Table 1; Figure 2A). However, this pattern characterized only females, while there were no differences between the five categories for males (Table 3; Figures 2B). Moreover, post-hoc tests (Table 3) revealed that the total number of strategies was higher for males with no contact with minors (*M* = 1.59, *SD* = 1.90, 95% CI [1.11–2.06]) compared to females (*M* = 1.09, *SD* = 1.50, 95% CI [0.81–1.35]) and to participants having contact with minors (males: *M* = 1.08, *SD* = 1.33, 95% CI [0.82–1.36]; females: *M* = 1.31, *SD* = 1.61, 95% CI [1.16–1.46]).

**Table 3:** Chi square (with degrees of freedom) and level of significance for significant effects and interactions, and key Bonferroni tests and effect sizes, for the GLMM for the survey about the families of coping strategies concerning the news about wars.

Significant effects or interactions	*χ^2^ (df)*	*p*	Bonferroni post-hoc comparisons	*z*	*p*	*d*
Family	42.04 (4, 141)	< 0.001	Problem solving vs. Information seeking	0.22	n.s	0.04
			Problem solving vs. Self-reliance	−3.25	0.012	−0.48
			Problem solving vs. Social support	−4.83	< 0.001	−0.67
			Problem solving vs. Accommodation	−3.85	0.001	−0.55
			Information seeking vs. Self-reliance	−3.34	0.009	−0.52
			Information seeking vs. Social support	−4.83	< 0.001	−0.71
			Information seeking vs. Accommodation	−3.91	0.001	−0.58
			Self-reliance vs. Social support	−1.51	n.s.	−0.19
			Self-reliance vs. Accommodation	−0.51	n.s.	−0.07
			Social support vs. Accommodation	1.05	n.s	0.13
Gender × Contact with minors	6.64 (1, 141)	0.010	Males: No contact with minors vs. Contact with minors	1.79	n.s.	0.37
			Females: No contact with minors vs. Contact with minors	−1.97	n.s.	−0.29
			No contact with minors: Males vs. Females	2.64	0.050	0.54
			Contact with minors: Males vs. Females	−0.83	n.s.	−0.13
Gender × Family	25.32 (4, 141)	< 0.001	Males: Problem solving vs. Information seeking	−0.01	n.s.	< −0.001
			Males: Problem solving vs. Self-reliance	0.34	n.s.	< 0.001
			Males: Problem solving vs. Social support	−1.32	n.s.	< −0.001
			Males: Problem solving vs. Accommodation	−1.82	n.s.	< −0.001
			Males: Information seeking vs. Self-reliance	0.34	n.s.	< 0.001
			Males: Information seeking vs. Social support	−1.32	n.s.	< −0.001
			Males: Information seeking vs. Accommodation	−1.82	n.s	< −0.001
			Males: Self-reliance vs. Social support	−1.66	n.s.	< −0.001
			Males: Self-reliance vs. Accommodation	−2.15	n.s.	< −0.001
			Males: Social support vs. Accommodation	−0.51	n.s.	< −0.001
			Females: Problem solving vs. Information seeking	−0.32	< 0.001	< −0.001
			Females: Problem solving vs. Self-reliance	−6.03	< 0.001	< −0.001
			Females: Problem solving vs. Social support	−6.16	0.006	< −0.001
			Females: Problem solving vs. Accommodation	−3.79	< 0.001	< −0.001
			Females: Information seeking vs. Self-reliance	−5.69	< 0.001	< −0.001
			Females: Information seeking vs. Social support	−5.79	0.008	< −0.001
			Females: Information seeking vs. Accommodation	−3.74	n.s.	< −0.001
			Females: Self-reliance vs. Social support	−0.12	n.s.	< −0.001
			Females: Self-reliance vs. Accommodation	2.42	n.s.	< 0.001
			Females: Social support vs. Accommodation	2.55	n.s.	<0.001
			Problem solving: Males vs. Females	2.62	n.s.	<0.001
			Information seeking: Males vs. Females	2.73	n.s.	<0.001
			Self-reliance: Males vs. Females	−2.31	n.s.	<−0.001
			Social support: Males vs. Females	−0.71	n.s.	< −0.001
			Accommodation: Males vs. Females	1.63	n.s.	<0.001

On the whole, the responses referred more to adaptive rather than maladaptive coping strategies. Therefore, we decided to include in the first pamphlet only examples of adaptive tips. This choice was also supported by the fact that elaborating persuasive messages including negations is cognitively more demanding vs. elaborating those without them, given the longer processing time and the higher rates of errors compared to affirmative statements (Kaup et al., 2007), and that for inserting maladaptive strategies we should have used formulations with negations.

To further define the contents of the first pamphlet, a pool of five experts in psychology and general sociology selected four examples for each family. To make the pamphlet easy to consult, following a previous campaign (Raccanello et al., 2020), we divided the strategies into three subsections, related to the three basic needs to which they refer, using more intelligible labels: *Look for solutions*, *Seek and give support*, and *Understand what is important,* respectively, for the need for competence, relatedness, and autonomy. Each subsection reported two families of strategies, again renamed (Table 1; Figure 1A).

For developing the contents of the second pamphlet, the same pool of experts primarily considered those of the previous one. We used the same structure in the part concerning coping tips, utilizing also the same labels for both subsections and strategy families. The experts selected the strategies to be inserted by (a) adapting examples from the first pamphlet, and (b) coding strategies from PFA manuals (NCTSN and NCPTSD, 2006; World Health Organization et al., 2011) according to Zimmer-Gembeck and Skinner’s (2011) classification. After discussion, the experts chose four examples for each category (Figure 1B).

### Testing the campaign message

We tested the comprehensibility, the usability, and the utility of the pamphlets.

As for the first pamphlet, the 40.7% of the participants considered it very comprehensible, the 42.6% comprehensible, and the 16.7% moderately comprehensible. Concerning usability, the pamphlet consultation was evaluated very easy by the 29.6%, easy by the 51.9%, and moderately easy by the 18.5%. Moreover, the 15.1 and 20.4% perceived, respectively, the message as very useful for themselves and for children/adolescents, the 35.8 and 48.2% as useful, the 45.3 and 27.8% as moderately useful, and the 3.7 and 3.7% as a little useful.

The second pamphlet was judged very comprehensible by the 33.3% of the sample, comprehensible by the 59.3%, and moderately comprehensible by the 7.4%. As concerns usability, the 35.2% considered it very easy to consult, the 57.4% easy, the 5.6% moderately easy, and the 1.8% a little easy. Furthermore, the pamphlet was evaluated by the 13.2 and 30.2% of the participants as very useful, respectively, for themselves and for children/adolescents, by the 45.3 and 45.3% as useful, by the 35.8 and 16.9% as moderately useful, and by the 3.8 and the 7.6% as a little useful; only a participant (i.e., the 1.9%) considered it no useful for him/herself.

### Monitoring the campaign dissemination

We monitored the dissemination of the campaigns considering indicators from the Google Analytics™.

The webpage of the first pamphlet from its publication to the 30^th^ June 2022 had 1,112 views. The 95.6% came from Europe (particularly, the 88.7% from Italy), the 3.1% from the Americas, the 1.1% from Asia, and the 0.2% from Oceania. As regards the traffic type, the 48.7% of the visitors came to the webpage through referrals, the 43.5% directly, and the 7.8% through organic search.

As for the second pamphlet, its webpage obtained 143 views from the publication to the 30^th^ June 2022. Concerning their location, most views derived from Europe (84.6%; specifically, the 71.3% from Italy), followed by the Americas (9.1%), Asia (3.5%), and Oceania (2.8%). Moreover, the traffic was referral for the 44.8%, direct for the 37.7%, and organic for the 17.5%.

## Discussion and conclusion

### Discussion of main findings

This community case study described how we developed two public communication campaigns targeting lay adults who have a role in caring for young people’s wellbeing in the context of armed conflicts.

The first step was to develop the messages for the campaigns. We adapted the three-part content of a previous public communication campaign about the COVID-19 pandemic (Raccanello et al., 2020). We provided basic information about the nature of the emergency (i.e., armed conflicts), considering as key sources the psychological literature on the effects of media exposure (Pfefferbaum et al., 2019) and PFA manuals (World Health Organization et al., 2011). The other sections operationalized the components of emotional competence (Denham, 1998). Basing on Ekman’s (1992, 1993) list of primary emotions, we drew the reader’s attention to a range of negative emotions that are typical consequences of traumatic events. We emphasized the need to restore positive feelings as a significant stage in developing resilience. As the theoretical basis for prescribing coping strategies, we used Zimmer-Gembeck and Skinner’s (2011) classification. When choosing specific content for the first pamphlet, we drew on the survey responses of an *ad hoc* sample of adults. The thematic content of the responses, together with the statistical analyses, enabled us also to explore the perceived salience of the different strategies when applied to exposure to war-related news. In this way we extended the existing literature. We found that the most frequently mentioned adaptive categories were social support and self-reliance (both associated with the need for relatedness; Deci and Ryan, 1985), and accommodation (need for autonomy). Information seeking and problem solving (need for competence) were seen as equally salient by males but less salient by females. We could speculate that this is linked, in line with stereotypical cultural roles, with females’ higher attention to relationships and care, together with a salient need for autonomy when compared to males. In addition, males who had no contact with minors reported a higher number of strategies overall. Because this result seems coherent with cultural stereotypes, we could presume that interest in news about armed conflicts is higher for males, in the absence of personal or professional reasons for focusing on such topics. Nevertheless, future research should explore these speculations, maybe related to the gender imbalance in our sample. The least reported adaptive strategy was negotiation (need for autonomy). Not surprisingly, there were few mentions of maladaptive strategies—helplessness and escape (need for competence), and opposition (need for autonomy) being the ones mentioned. Our results were in line with published reports exploring the use of adaptive or maladaptive coping strategies in the context of natural disasters such as earthquakes or pandemics (Raccanello et al., 2021a,b, 2022a). For the second pamphlet, we relied both on the first pamphlet and on relevant guidelines for PFA (NCTSN and NCPTSD, 2006; World Health Organization et al., 2011). Finally, the second pamphlet included some recommended ways for adult helpers to support their own wellbeing. An issue of importance given the established links between young people’s mental state and that of their parents (Dimitry, 2012; Slone and Mann, 2016; Vossoughi et al., 2018; Aghajafari et al., 2020; Eltanamly et al., 2021; Jin et al., 2021).

To establish the usefulness of the pamphlet content, we conducted online surveys using two *ad hoc* samples. The data provided indications that the content could be understood, implemented, and would be useful. However, more in-depth research should be conducted to understand why some participants gave relatively low ratings for one or more of the three criteria. For example, it would be interesting to understand which aspects of the pamphlets determined a low comprehensibility and usability (e.g., linguistic formulations, graphic format, quality of drawings, dimensions of typing, etc.) or a low utility (e.g., thoughts about more useful tips, low interest for news about wars, low emotional involvement in current world events, etc.).

We monitored the dissemination of the campaigns from the release of the pamphlets to the end of June 2022. Google Analytics™ data showed substantially more views for the first pamphlet compared with the second. One reason for this could be that the second pamphlet was mainly distributed from the hubs established to welcome Ukrainian refugees. The webpages of both pamphlets were viewed mostly within European countries (particularly Italy). Finally, the traffic to both webpages was mediated by other referral websites or discovered directly; however, a relatively small number of visitors accessed the pamphlets by searching from their browser. The monitoring is still in progress.

### Limitations and future directions

This study suffers from some limitations. First, we could not test the efficacy of the campaigns applying evidence-based standards (Flay et al., 2005; Gottfredson et al., 2018), for example comparing their effects for an experimental group exposed to the pamphlets vs. a not-exposed control group. Considering the challenges of emergencies, researchers are currently developing alternative designs for overcoming the limitations of such contexts, such as matching participants on the bases of propensity scores or randomizing groups rather than individuals (Grolnick et al., 2018). Second, we could not gather data from our target population, i.e., children and adolescents, nor we could monitor the long-term efficacy of our interventions. Future research could build alliances with territory partners and community networks to identify innovative ways to collect information about their impact (Grolnick et al., 2018). Third, the psychoeducational materials that we developed covered only some of the relevant contents for emotionally supporting people in such situations, therefore they are not exhaustive. Beyond focusing on emotions and coping strategies, future materials could refer, for example, to attitudes and behaviors, following traditional persuasion models (Fishbein and Ajzen, 1975), or to threat severity and vulnerability together with self-efficacy, as detailed by the protection motivation theory to promote health (Floyd et al., 2000).

### Conclusion

Assisting adults in supporting children and adolescents exposed to wars and other disasters is a fundamental step to promote young people’s resilience in face of such traumatic events. Future research should examine deeper the impact of similar campaigns, developing innovative tools for studying their effects within a variety of emergency contexts.

## Data availability statement

The raw data supporting the conclusions of this article will be made available by the authors to any qualified researcher upon request.

## Ethics statement

The studies involving human participants were reviewed and approved by the Ethical Committee of the Department of Human Sciences, University of Verona. The participants provided their written informed consent to participate in this study.

## Author contributions

GV and DR contributed to conception and design of the study, and wrote the first draft of the manuscript. GV, RB, ER, CL, RH, and DR contributed to conception and design of pamphlets. GV, RB, ER, and DR organized the databases. RB, ER, CL, and RH wrote sections of the manuscript. All authors contributed to the article and approved the submitted version.

## Conflict of interest

RH was employed by Environmetrics Pty Ltd.

The remaining authors declare that the research was conducted in the absence of any commercial or financial relationships that could be construed as a potential conflict of interest.

## Publisher’s note

All claims expressed in this article are solely those of the authors and do not necessarily represent those of their affiliated organizations, or those of the publisher, the editors and the reviewers. Any product that may be evaluated in this article, or claim that may be made by its manufacturer, is not guaranteed or endorsed by the publisher.

## Footnotes

^1^ https://www.hemot.eu/risorse-guerre/

^2^ https://www.hemot.eu

^3^ https://www.hemot.eu/2022/03/28/news-about-wars-psychological-tips-for-children-and-adolescents-emotions/

^4^ https://www.hemot.eu/2022/05/19/psychological-tips-victims-wars/
